# Medical Items

**Published:** 1908-12

**Authors:** 


### MEDICAL ITEMS

---

As the cold damp winds of fall chill the skin, more of the work of elimination is thrown upon the kidneys. It is not always the function of the kidney can be adjusted to this increased demand, and imperfect elimination of waste products results.

This autotoxic state gives rise to such conditions as rheumatism, tonsillitis, neuralgia, catarrhal bronchitis, with or without asthma, winter eczema and pruritis, catarrhal rhinitis, and many other less distinctly defined conditions.

The best results in treatment are to be had from establishing thorough renal elimination. Nothing accomplishes this so promptly and so effectually as Alkalithia in teaspoonful doses, in half a glass of water, every four hours, and a cure follows the removal of the cause.

---

It is generally conceded that cod liver oil is to be considered more as food than as medicine. Potter says: "Cod liver oil is the most easily digestable of fats, penetrating animal membranes with comparative ease, hence entering the lacteal vessels readily and appearing to carry with it the oily and nitrogenous elements of the food."

But every physician understands the uses of cod liver oil, what concerns him most is, to have it in such form that his patient will take it and enjoy taking it, for after all, it is the continued use and assimilation of this valuable nutrient that brings the best results.

The physician reader will be glad to know that there is now within range of his prescription pad, an emulsion of cod liver oil, that a prominent physician characterizes as follows: "One of the most elegant preparations of modern pharmacy in EMULSION OLEI MORRHUAE (Cloftlin). Don't make the mistake of looking on this as only one more emulsion, but investigate its unique merits."

We are quite sure, that nothing would please its manufacturers better than to have every physician reader of this Journal make use of their perogative, to investigate by careful clinical test, the good qualities of EMULSION CLOFTLIN We are informed that liberal samples and full descriptive matter will be sent express prepaid, to all registered physicians, who will send their name and express office address to The Cloftlin Chemical Co., 75-77 Cliff street, New York.

*The Cloftlin Chemical Co., 75 Cliff street, City*:

Gentlemen: At your request, we purchased a package of the medicinal preparation called Emulsion Cloftlin, at a pharmacy on Broadway, in this City, and, upon careful analysis of the same, we find it contains the following medicinal ingredients in the quantities below stated; to-wit:

Cod Liver Oil, by weight, 51.05 per cent.

Glycerine, by weight, 14.41 per cent.

Calcium Hypophosphite, by weight, 1.57 per cent—7 grains per fluid oz.

Manganese Hypophosphite, by weight, 0.66 per cent.—3 grains per fluid oz.

This is equivalent, in the case of the cod liver oil, to fully 50 per cent. by volume, and of the glycerine, 10 per cent.

Very truly yours,

LEDERLE LABORATORIES.

October 27, 1906.

